# Morphological and functional correlates of vestibular synaptic deafferentation and repair in a mouse model of acute-onset vertigo

**DOI:** 10.1242/dmm.039115

**Published:** 2019-07-15

**Authors:** Raphaelle Cassel, Pierrick Bordiga, Julie Carcaud, François Simon, Mathieu Beraneck, Anne Le Gall, Anne Benoit, Valentine Bouet, Bruno Philoxene, Stéphane Besnard, Isabelle Watabe, David Pericat, Charlotte Hautefort, Axel Assie, Alain Tonetto, Jonas Dyhrfjeld-Johnsen, Jordi Llorens, Brahim Tighilet, Christian Chabbert

**Affiliations:** 1 Aix Marseille Université, CNRS, UMR 7260, Laboratoire de Neurosciences Sensorielles et Cognitives - Equipe Physiopathologie et Thérapie des Désordres Vestibulaires, Marseille, 13000 France; 2 Integrative Neuroscience and Cognition Center, UMR 8002, CNRS, 75006 Paris, France; 3 Sorbonne Paris Cité, Université Paris Descartes, 75006 Paris, France; 4 INSERM U1075, Caen, France; 5 Hôpital Lariboisière, 75010 Paris, France; 6 Aix-Marseille Université, CNRS, Centrale Marseille, FSCM (FR1739), PRATIM, Marseille, 13000 France; 7 Sensorion, Montpellier, 34000 France; 8 Universitat de Barcelona, 08907 Barcelona, Spain

**Keywords:** Vestibule, Synapses, Excitotoxicity, Plasticity, Vestibular disorders

## Abstract

Damage to cochlear primary afferent synapses has been shown to be a key factor in various auditory pathologies. Similarly, the selective lesioning of primary vestibular synapses might be an underlying cause of peripheral vestibulopathies that cause vertigo and dizziness, for which the pathophysiology is currently unknown. To thoroughly address this possibility, we selectively damaged the synaptic contacts between hair cells and primary vestibular neurons in mice through the transtympanic administration of a glutamate receptor agonist. Using a combination of histological and functional approaches, we demonstrated four key findings: (1) selective synaptic deafferentation is sufficient to generate acute vestibular syndrome with characteristics similar to those reported in patients; (2) the reduction of the vestibulo-ocular reflex and posturo-locomotor deficits mainly depends on spared synapses; (3) damaged primary vestibular synapses can be repaired over the days and weeks following deafferentation; and (4) the synaptic repair process occurs through the re-expression and re-pairing of synaptic proteins such as CtBP2 and SHANK-1. Primary synapse repair might contribute to re-establishing the initial sensory network. Deciphering the molecular mechanism that supports synaptic repair could offer a therapeutic opportunity to rescue full vestibular input and restore gait and balance in patients.

## Abstract (precis)

**Summary:** The molecular rearrangements of the synaptic proteins that accompany the deafferentation and subsequent reafferentation of the inner ear sensors following an excitotoxic insult are demonstrated for the first time.

## INTRODUCTION

Acute unilateral vestibulopathy (AUV) has well-defined clinical features, including severe vertigo attacks, static and dynamic imbalances, spontaneous nystagmus, nausea and vomiting (Strupp et al., 2004; Strupp and Magnusson, 2015). Information is still lacking on the aetiology, the pathophysiological processes involved and the specific anatomical substrate that is affected, as well as on the correlation between tissue lesions and the occurrence of the various symptoms that constitute the vestibular syndrome. The neuroinflammatory hypothesis has been largely proposed based on the suspicion of inflammatory foci along the different branches of the vestibular nerve (Ruttin, 1909; Rauch, 2001; Richard and Linthicum, 2012). With the evolution of methods for vestibular functional evaluation, questions have been increasingly raised regarding the precise anatomical locations of the lesions that cause AUV symptoms. Recently, the hypothesis of an intralabyrinthine origin has been proposed (Hegemann and Wenzel, 2017).

The area of contact between the inner ear hair cells and the fibres forming the eighth cranial nerve is considered the most vulnerable area of the inner ear (Liberman and Kujawa, 2017). In the cochlea, selective damage to the primary auditory synapses has been implicated in acquired sensorineural hearing loss. A recent histopathological quantitative analysis of the aged human inner ear confirmed previous observations in animals, which suggested that cochlear synaptopathy and the degeneration of cochlear nerve peripheral axons (despite near-normal hair cell populations) might be an essential component of human age-related hearing loss (Viana et al., 2015). In the vestibule, direct evidence of primary synapse damage under pathological conditions or as a result of ageing is lacking. It has been proposed, however, that the impairment of primary synapses might be involved in vestibular neuritis, labyrinthitis, vertigo of ischaemic origin and Menière disease (Rauch, 2001; Halmagyi et al., 2010; Foster and Breeze, 2013).

Aiming to decipher the correlation between selective damage to the primary vestibular synapses and the occurrence of AUV symptoms, we induced unilateral vestibular excitotoxic lesions in young adult mice through the transtympanic administration of kainic acid (TTK), as previously reported (Cassel et al., 2018). Using light and transmission electron microscopy (TEM), we performed a thorough histological analysis of the damage within the inner ear at different time points, over a period spanning the first hours following lesion induction and up to 3 weeks later. To quantitatively evaluate the proportion of deafferented synapses following the TTK administration, we performed immunostaining for CtBP2 (RIBEYE) and SHANK-1, two synaptic proteins present, respectively, in the inner ear hair cells presynaptic ribbons (Khimich et al., 2005) and in the glutamatergic postsynaptic density (PSD) of their cognate afferent fibres (Huang et al., 2012; Braude et al., 2015). In parallel, video-oculography was used to quantify the occurrence of the nystagmus, as well as the angular vestibulo-ocular reflex (aVOR) and maculo-ocular reflex (MOR), at each time point following the induction of the vestibular excitotoxic lesion, according to previously described protocols (Beraneck and Idoux, 2012; Carcaud et al., 2017). Alterations in gait and balance and in the general behaviour of the animals were also evaluated by specific behavioural testing following previously described methods (Cassel et al., 2018). The two types of functional evaluation were also performed in adult mice after unilateral vestibular neurectomy (UVN) to compare the functional consequences of transient versus permanent unilateral vestibular loss.

The parallel analysis of the temporal evolution of vestibular histological lesions and the resulting functional alterations allows for the evaluation of the specific contribution of peripheral synaptic plasticity to the occurence of the vertigo syndrome. The data demonstrate that synaptic deafferentation is sufficient to produce vertigo symptoms and its associated deficits with a time course of development and recovery that resembles the clinical situation. In turn, the study highlights the primary vestibular synapses as areas that are highly exposed to excitotoxic damage and sets the stage for future pharmacological studies on the development of efficient therapeutic countermeasures.

## RESULTS

### Induction of peripheral vestibular excitotoxic lesions

**Fig. 1.**
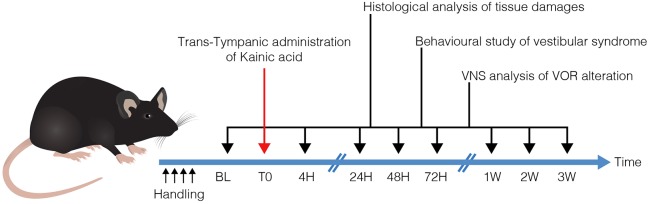
**Operational diagram used in the study.** Following TTK (T0), histological and functional analyses were performed at each of the indicated time points. Histological analysis of inner ear tissues was performed using TEM. Assessment of posturo-locomotor alterations was performed using specific behavioural testing. aVOR alterations were measured using video-oculography. BL, base line; VNS, videonystagmoscopy.

TTK administration was performed in adult mice of both sexes, according to a recently described method (Cassel et al., 2018). Histological and functional evaluations were performed on groups of naive and lesioned animals at several time points (4 h, 24 h, 48 h, 72 h, 1 week, 2 weeks and 3 weeks) following vestibular excitotoxicity induction (Fig. 1).

### TTK administration induces the transient deafferentation of primary vestibular synapses

**Fig. 2.**
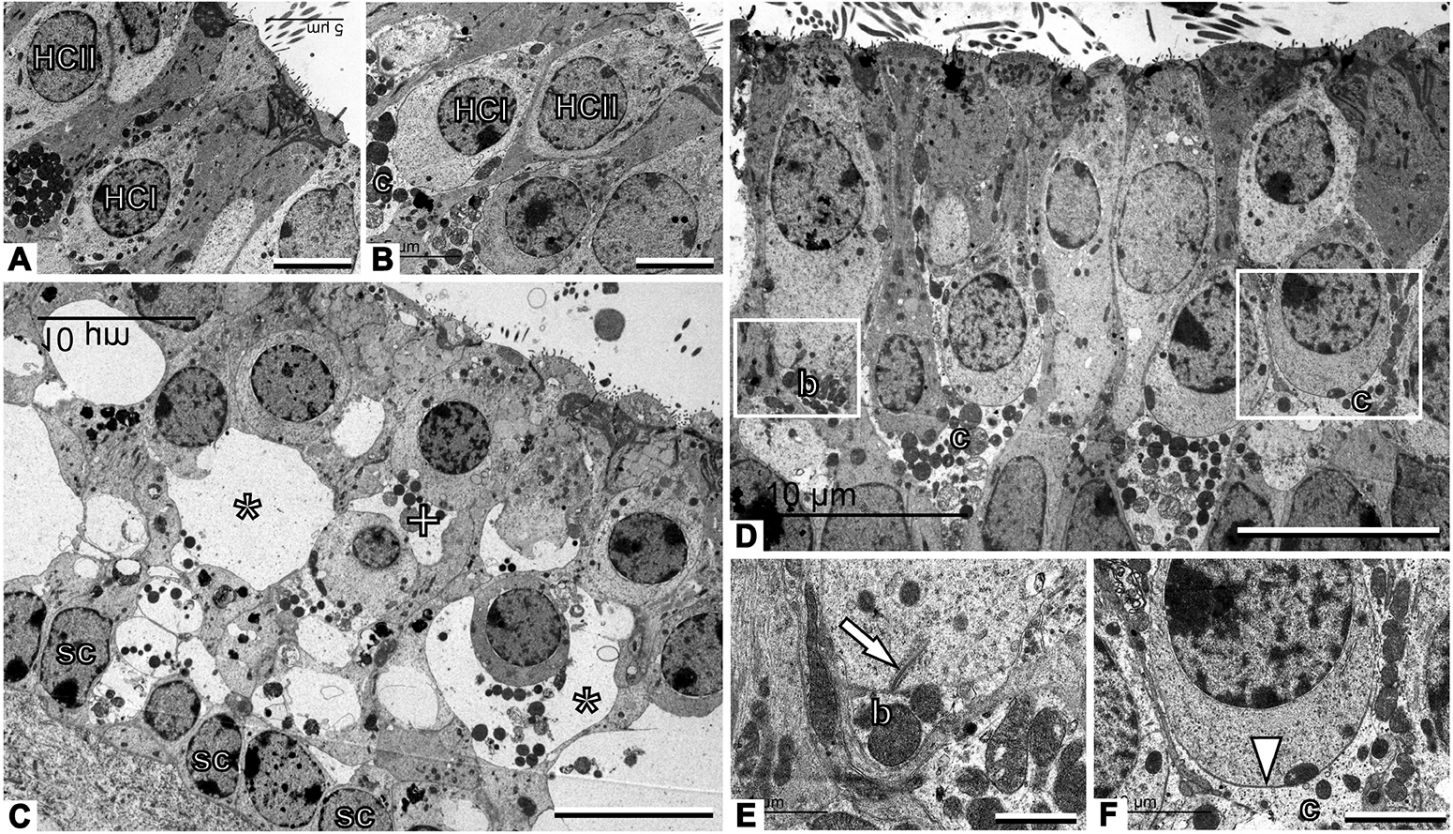
**TEM observation of vestibular primary synapses following TTK in the utricle.** (A,B) Sham vestibular epithelium. Type I hair cells (HCI) are recognised by their amphora-like shape and the calyx afferent (c) encasing the cell body. Type II hair cells (HCII) have a more cylindrical shape and no calyx afferent. (C) Afferents are greatly swollen and the structure of the epithelium is completely distorted 4 h after TTK. Some swollen afferents probably corresponded to calyx terminals (*), whereas others are more likely to correspond to bouton terminals (+). Note the row of supporting cell nuclei (sc) just above the basal membrane of the epithelium. (D) The epithelium has grossly recovered a control-like appearance 1 week after TTK. (E) Higher magnification of the area marked with the left box in D, showing control-like ultrastructure including a presynaptic ribbon (arrow) in a type II hair cell facing a postsynaptic bouton afferent (b). (F) Higher magnification of the area marked with the right box in D, showing a contact between a type I hair cell and the calyx afferent contacting it. The arrowhead points to the calyceal junction, a prominent characteristic of this contact in healthy and mature epithelia. Sample: *n*=3 in each group. Scale bars: 5 µm (A,B), 10 µm (C,D), 1 µm (E) and 2 µm (F).

Light microscopy and TEM examination of fixed tissue from the vestibular endorgans revealed histological features characteristic of excitotoxic damage in both the utricle (Fig. 2 and Fig. S1) and the crista ampullaris (Fig. S2). Utricles from the sham-injected ears (which received vehicle without kainic acid) and contralateral ears showed normal ultrastructural features (Fig. 2A,B). By 4 h after TTK administration, most of the afferent nerve terminals of the Scarpa's ganglion neurons were highly swollen, with large vacuoles evident along the hair cell membranes throughout the sensory epithelia (Fig. 2C). Many hair cells were distorted owing to the size of the swollen nerve terminals and were sometimes no longer identifiable as type I or type II hair cells with regard to their morphology. Supporting cells were also squeezed, although their nuclei remained in the normal position. Membrane disruptions in damaged afferent terminals were often observed (Fig. S1). These histopathological features of excitotoxic damage were no longer visible in the utricle 1 week after the onset of the lesion, and the two types of hair cells were easily recognisable by their respective calyx and bouton afferent contacts and were morphologically distinct from the supporting cells (Fig. 2D). A detailed examination revealed normal ultrastructural features in both bouton (Fig. 2E) and calyx (Fig. 2F) afferents; however, some of the calyx endings showed discontinuity or a fragmented appearance (Fig. S1). In the cristae (Fig. S2), substantial swelling of afferents was observed 4 h after TTK administration, but this swelling was concentrated in the central part of the sensory epithelium. As in the utricle, the lesions in the crista were largely repaired 1 week after TTK administration. Together, these observations confirmed that, as previously reported in the rat (Brugeaud et al., 2007; Dyhrfjeld-Johnsen et al., 2013; Gaboyard-Niay et al., 2016), TTK administration in adult mice induced transient and specific damage to the synaptic contacts between the vestibular hair cells and their cognate nerve fibres arising from primary vestibular neurons.

**Fig. 3.**
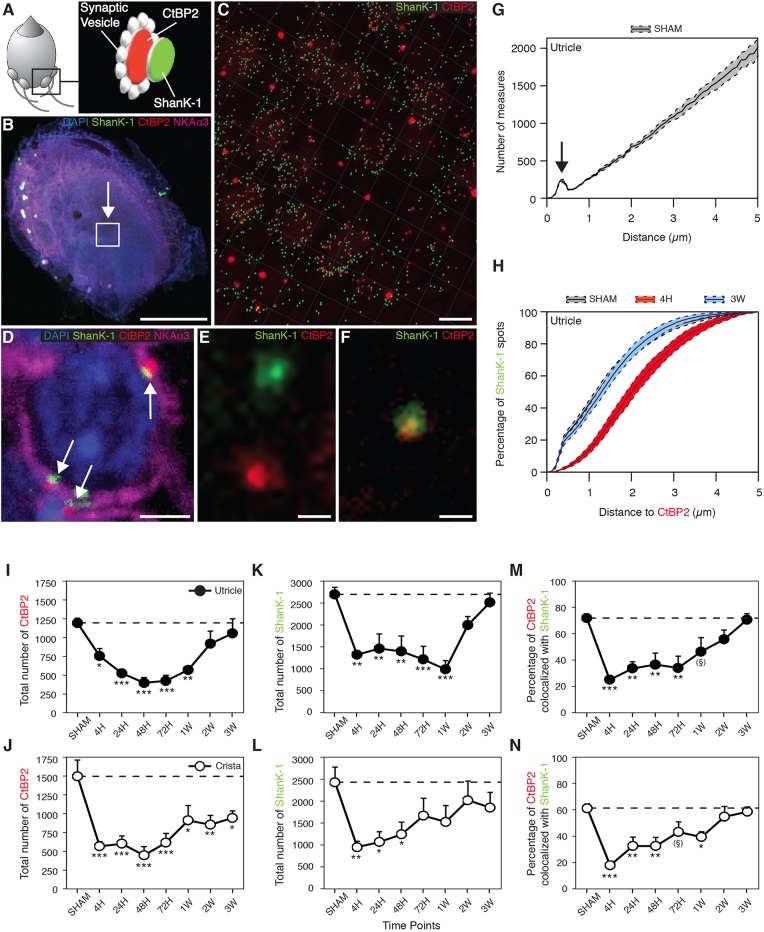
**Evaluation of synaptic protein expression following TTK administration.** (A) Schematised vestibular bouton synapses with the respective locations of immunostained CtBP2 (red) and SHANK-1 (green) proteins. (B) Observation field (arrowed box) of 4.5e^−03^ mm^2^, which includes about 75-100 hair cells in the centre of a utricle. (C) CtBP2 and SHANK-1 fluorescent spots semi-automatically detected within the observation field using IMARIS software. (D) Characteristic expression of fluorescent spots at a calyx terminal (white arrows). (E) Distant and (F) colocalised spots at high magnification. (G) Repartition of the distances between all CtBP2 and SHANK-1 fluorescent spots highlighting a specific subpopulation of spots, the interdistances of which are located within 1 μm (arrow). Images in B-D,F,G: control utricles. (H) Cumulative distances between CtBP2 and SHANK-1 before (sham), 4 h and 3 weeks after TTK application. Note that the gray sham data overlap with the 3 week data. (I-N) Expression of CtBP2 (I,J), SHANK-1 (K,L) and the percentage of their colocalisation (M,N) at each time point before and after TTK application in both the utricle (black circles; *n*=6 for 48 h, 72 h and 2 weeks; *n*=5 for sham, 24 h and 3 weeks; *n*=4 for 4 h and 1 week) and crista ampullaris (white circles; *n*=6 for 4 h, 72 h and 3 weeks; *n*=5 for sham, 24 h, 48 h, 1 week and 2 weeks). The ribbon counts were normalised relative to the observation field described in the Materials and Methods section. Results are expressed as mean±s.e.m. Dotted lines represent sham values. ANOVA followed by Dunnett post-hoc analyses were performed to compare each time point to the sham: *P<0.05; **P<0.01; ***P<0.001; ^(§)^*P*=0.6. Scale bars: 200 µm (B), 5 µm (C), 2 µm (D) and 0.5 µm (E,F).

To quantitatively assess the magnitude of synapse deafferentation following TTK administration, fixed tissue from the vestibular endorgans was immunostained for CtBP2 (a component of the inner-ear hair cells presynaptic ribbons) and SHANK-1 (a scaffold protein of the glutamatergic postsynaptic density of excitatory synapses). Using 3D confocal microscopy acquisition and image analysis, we extracted the position of each CtBP2-positive fluorescent spot and evaluated its distance from the nearest neighbouring SHANK-1-positive fluorescent spot within observation fields of 4.5e^−03^ mm^2^, chosen in the central part of both the utricle (extra-striolar region) and the hemi-crista. Figure 3 illustrates the CtBP2 and SHANK-1 expression at a schematically represented vestibular bouton synapse (Fig. 3A), the location of a selected observation field in the utricular macula (Fig. 3B), the fluorescent spots detected using *z*-stack compilation 3D reconstruction with IMARIS software in a representative observation field (Fig. 3C) and the characteristic expression of the CtBP2 and SHANK-1 fluorescence at a calyx synapse (Fig. 3D), both distantly localised and colocalised (Fig. 3E,F). The analysis of the distances between all CtBP2- and SHANK-1-labelled pre- and postsynaptic structures revealed a subpopulation of fluorescent spots separated by 1 μm or less (Fig. 3G). A previous evaluation of the distances between presynaptic dense bodies and the postsynaptic densities in the rodent vestibular endorgans (Sadeghi et al., 2014) suggests that this population corresponds to pre- and postsynaptic proteins at intact primary vestibular synapses. In the sham vehicle-injected mice, 40% of the distances between pre- and postsynaptic proteins in the utricle were ≤1 μm and 75% were ≤2 μm (Fig. 3H). By 4 h after vestibular TTK lesion, only 15% of the co-labelling was ≤1 μm apart, 50% was ≤2 μm apart and 80% was ≤3 μm apart. Conversely, 3 weeks after the induction of vestibular lesion, 40% and 80% of the co-labelling were once again found to be ≤1 μm apart and ≤2 μm apart, respectively. Together, these observations demonstrated that, after the induction of an excitotoxic synaptic lesion, the distance between the pre- and postsynaptic proteins increased significantly, probably signifying the deafferentation of vestibular hair cells. This deafferentation spontaneously recovered in the weeks after injury, as the expression pattern of CtBP2 and SHANK-1 returned to the pre-lesion state after 3 weeks.

The observation of the pre- and postsynaptic protein expression following the induction of the excitotoxic lesion demonstrated a dramatic reduction in both the expression of CtBP2 (*F*_7_=7.263, *P*<0.001) and SHANK-1 (*F*_7_=5.445, *P*<0.001) immediately after lesion induction (Fig. 3I-L). In the utricle, the proportion of CtBP2 fluorescent spots decreased from 100±5% (*n*=5) to 63±9% (*P*<0.05; *n*=4) and 33±6% (*P*<0.001; *n*=6) 4 h and 48 h after TTK administration, respectively (Fig. 3I). In parallel, the expression of SHANK-1 decreased from 100±6% (*n*=5) to 49±2% (*P*<0.01; *n*=4) and 52±13% (*P*<0.01; *n*=6) at 4 h and 48 h, respectively (Fig. 3K). In the crista, the proportion of CtBP2 spots decreased (*F*_7_=5.911, *P*<0.001) from 100±14% (*n*=5) to 38±4% (*P*<0.001; *n*=6) and 30±8% (*P*<0.001; *n*=5) 4 h and 48 h after TTK administration, respectively (Fig. 3J). Similarly, the expression of SHANK-1 significantly decreased (*F*_7_=2.293, *P*<0.05) from 100±14% (*n*=5) to 39±5% (*P*<0.01; *n*=6) and 51±11% (*P*<0.05; *n*=5) by 4 h and 48 h, respectively (Fig. 3L). Beginning at 1 week, we observed an increase in the levels of the two synaptic proteins in the two vestibular endorgans. By 3 weeks after the induction of the excitotoxic lesion, CtBP2 and SHANK-1 expression in the utricle recovered to 88±16% (*n*=5) and 93±8% (*n*=5) of the initial expression levels, respectively. At this time point, the expression of these two proteins was no longer significantly different from that in the sham group. CtBP2 and SHANK-1 expression in the crista recovered to 63±6% (*n*=6) and 76±14% (*n*=6) of the initial expression levels, respectively. Although SHANK-1 expression was not significantly different from that in the sham group, CtBP2 expression was still significantly reduced (*P*<0.01) (Fig. 3J,L).

We also quantified the percentage of colocalised CtBP2 and SHANK-1 (i.e. the percentage of CtBP2- and SHANK-1-positive fluorescent spots less than 1 μm apart). Following injury, in the utricle there was a reduction in the percentage of colocalisations (*F*_7_=5.794, *P*<0.001): from 72±2% to 25±3% (*P*<0.001) and 37±9% (*P*<0.01) between 4 h and 48 h after TTK administration, respectively. This observed reduction was followed by an increase to 46±11% at 1 week (*P*=0.06) and a recovery to the pre-lesional level at 2 and 3 weeks (no statistically significant difference; Fig. 3M). In the crista, the percentage colocalisation was reduced (*F*_7_=7.072, *P*<0.001) from 61±3% to 18±3% at 4 h after administration (*P*<0.001), followed by an increase up to 43±8% and 39±4% at 72 h and 1 week (*P*=0.06 and *P*<0.05, respectively). From 2 and 3 weeks onwards, the percentage colocalisation again increased to reach a near pre-lesion state (no statistically significant difference; Fig. 3N). The gross values of CtBP2 and SHANK-1 expression are shown in Table 1. Together, these observations indicated that the dynamic reafferentation of the vestibular mechanoreceptors occurred in the vestibular endorgans following excitotoxically induced deafferentation.

**Table 1.**
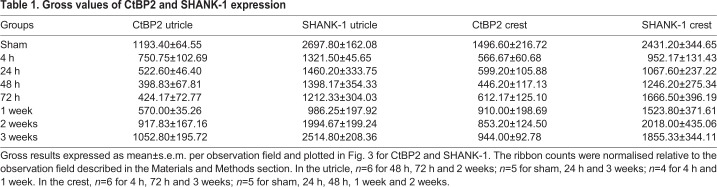
**Gross values of CtBP2 and SHANK-1 expression**

### Deafferentation of vestibular primary synapses evokes a transient alteration of the vestibulo-ocular reflex

**Fig. 4.**
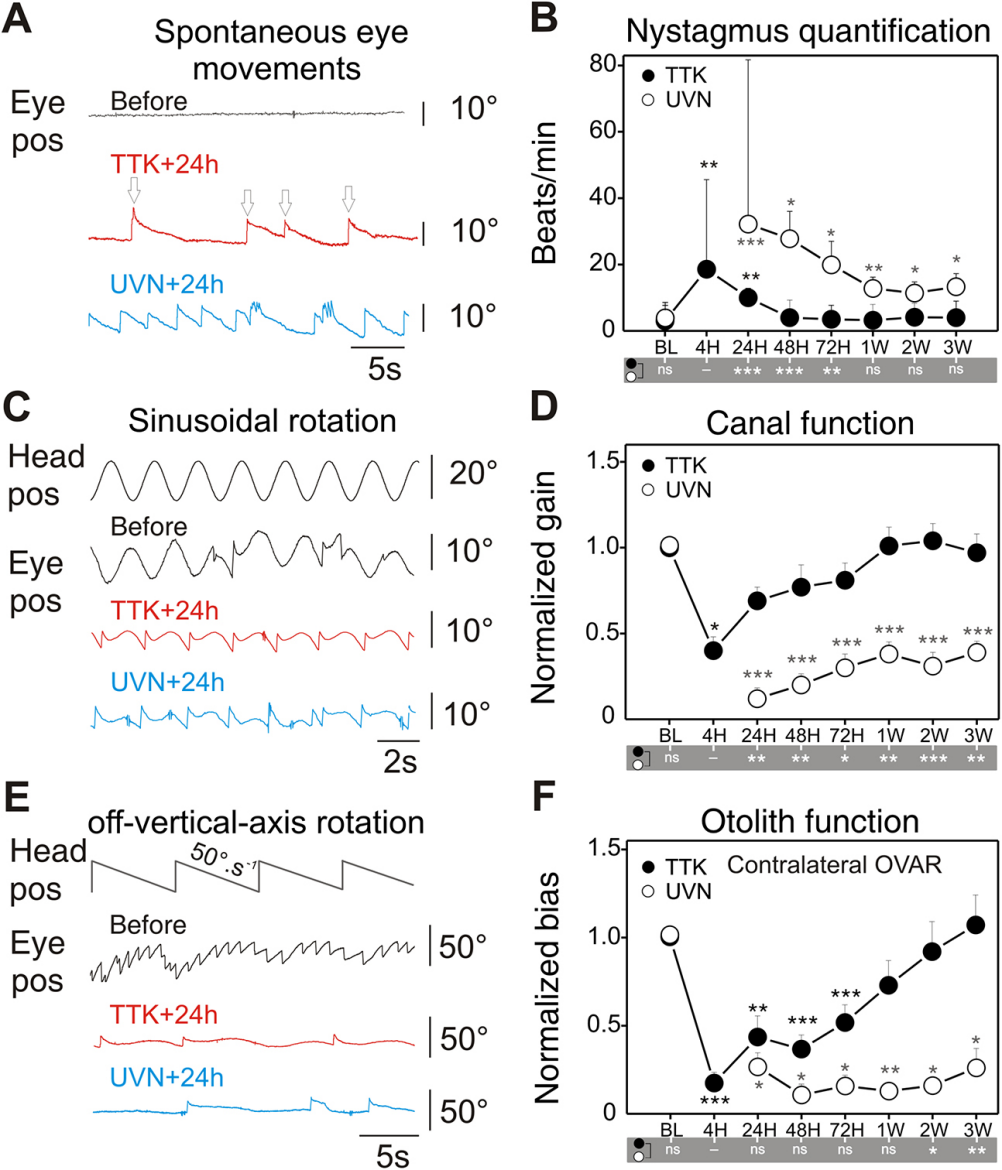
**Analysis of nystagmus, canal and otolith function alterations after TTK administration and UVN.** (A) Spontaneous eye movements were recorded in the dark before lesion and 24 h after TTK administration or UVN. Recordings show spontaneous eye drift (slow phase) and multiple quick phases (arrows) corresponding to pathological nystagmus. (B) Comparison of the nystagmus quantified from the TTK (*n*=11) and UVN (*n*=6) mice groups during the 3 weeks following the lesion. (C) Raw traces of eye movements observed during sinusoidal table rotation in yaw plane at 0.5 Hz and 30°/s sinusoidal rotation, before and 24 h after TTK or UVN. Note the reduction in amplitude of the eye movements and asymmetry in the response towards the ipsilesional side or contralateral rotation (here ipsi is up). (D) Quantification of the aVOR gain following TTK and UVN. To ease comparison, data are normalised to the pre-lesion values (raw values are reported in Table 2). (E) MOR were measured during off-vertical axis rotation at 50°/s. At 24 h after TTK and UVN, note the absence of the horizontal beating of the eye normally observed. (F) Quantification of the MOR bias following TTK and UVN. Data are normalised to the pre-lesion values. Results are presented as mean±s.e.m. If present, statistical significance is shown comparing each value to the base line (BL) value (repeated measures ANOVA and post-hoc Tukey tests, see Materials and Methods section). In the grey area under B, D and F, statistical differences between UVN and TTK values are reported (two- or three-way ANOVA and post-hoc Tukey tests, see Materials and Methods section). Statistics are represented as *P<0.05; **P<0.01; ***P<0.001; n.s., not specified. In all cases, the TTK population improved after 1 week and fully recovered after 3 weeks, whereas UVN mice remained pathological.

In the clinic, an imbalance of activity between opposite vestibules can be diagnosed by the presence of abnormal gaze stabilisation referred to as spontaneous vestibular nystagmus (SVN). In mice, the occurrence and characteristics of SVN were analysed at different time points following TTK administration (Fig. 4A,B). In the absence of vestibular stimulation and in complete darkness, 24 h after the lesion onset SVN was observed in 81% of the TTK-administered mice (*n*=9), but was not observed in any of the vehicle-treated sham mice (*n*=4). The slow phase of the nystagmus was always directed towards the ipsilesional side. The initial intensity of SVN (the number of beats/min) was variable between individuals at 4 h (mean±s.e.m. 18.6±11.1) and 24 h (mean±s.e.m. 10.0±2.7) and ranged from 1.5 to 24.7 beats/min. The frequency of SVN significantly decreased during the following 48 h in all TTK-administered mice and completely disappeared after 1 week in all but one mouse (Fig. 4C). When the same assessment was performed on mice that underwent UVN, SVN was observed in all lesioned animals at 24 h (it was not measurable at 4 h owing to skew deviation, i.e. post-lesion vertical ocular deviation); SVN occurred significantly more frequently (32.2±7; range 11-57; *F*_13_=10.567; *P*<0.001) in mice that underwent UVN than in the TTK-administered mice. SVN frequency also improved over the following days, but at a slower rate than in TTK-administered mice (intergroup differences are reported in Fig. 4 at the bottom of panels B, D and F). SVN was still observed in all (100%) animals after 1 week (12.8±3.4) and persisted in four animals (67%) beyond 3 weeks (13.3±3.9). The comparison of the TTK-administered mice and the UVN-lesioned mice therefore showed clear differences in both the initial intensity of SVN and in the dynamics of SVN recovery.

**Table 2.**
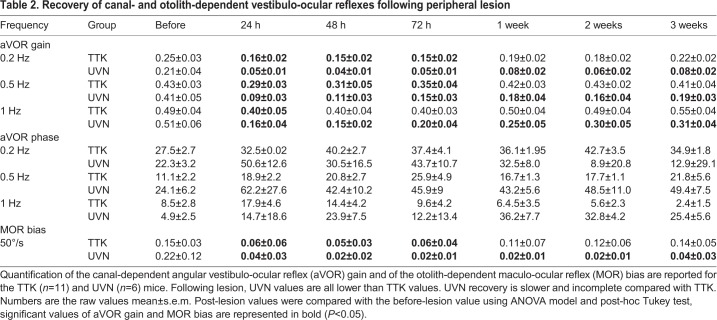
**Recovery of canal- and otolith-dependent vestibulo-ocular reflexes following peripheral lesion**

To assess semicircular canal function, video-oculography was performed in the dark during rotations in the horizontal plane (Fig. 4C,D). Sinusoidal rotations at frequencies between 0.2 and 1 Hz and 30°/s were performed, and the angular vestibulo-ocular reflex (aVOR) was quantified. At 4 h after TTK injection, the aVOR was significantly impaired but present in all mice. At all tested frequencies, the aVOR gain dropped to approximately 50% of the pre-lesion value: 32.9±6.0% for 0.2 Hz, 39.9±8.5% for 0.5 Hz and up to 50±10.6% for 1 Hz (Fig. 4E and Table 2). When the aVOR was tested again 24 h after lesion induction, we observed a significant recovery of the horizontal canal function normalised values: 66±7.3% for 0.2 Hz, 69±7.6% for 0.5 Hz and up to 83±9.3% for 1 Hz (raw values reported in Table 2). The recovery continued over the following days until full recovery was observed at all tested frequencies after 1 week (Fig. 4D). In the UVN group, the reduction in the aVOR was significantly more severe than that after TTK administration. By 24 h after lesion induction, the UVN gain remained decreased to 14±2.5%, 12±4.3% and 24±7.1% of the initial value for 0.2, 0.5 and 1 Hz, respectively (*F*_41_=18.818, *P*=0.944; *P*=0.007; *P*=0.001; compared with the TTK-administered group, using three-way ANOVA for time, frequency and lesion type)*.* After 3 weeks, the recovery of the aVOR gain remained incomplete in all UVN-lesioned mice, with a maximum recovery of approximately 45% of the initial value for all frequencies (Fig. 4D). Sham experiments were performed for both the TTK administration and UVN protocols (4 and 6 mice, respectively). Although the transtympanic sham injection did not trigger any vestibular symptoms or deficit, the UVN sham experiments (complete surgery including the opening of the bulla) led to a 33% immediate decrease in aVOR, which rapidly recovered over the following 2 days (data not shown). Overall, the comparison of the alteration of the aVOR in TTK-administered and UVN-lesioned mice showed differences in both the intensity of the initial symptoms and in the capacity for recovery, which was largely achieved after 1 week in the TTK-administered mice but remained incomplete after 3 weeks in the UVN-lesioned mice.

To assess otolithic function, the off-vertical axis rotation test was performed (Fig. 4E,F) as previously described (Beraneck and Idoux, 2012; Emptoz et al., 2017; Romand et al., 2013). Before lesion induction, normal MORs with clear compensatory eye movements were observed in all animals (Fig. 4E). After lesion induction, the MOR was analysed exclusively during rotation towards the non-lesioned side to prevent the confounding of spontaneous vestibular nystagmus and MORs. At 4 h after TTK injection, the MOR completely disappeared such that, in most instances, only the spontaneous nystagmus caused by the lesion was observed during the MOR test. During the following days, MORs improved but remained significantly impaired at 24 h, 48 h and 72 h (*F*_6_=5.940, *P*=0.004; *P*=0.008; *P*=0.024). Finally, after 1 week the MOR returned to the normal range and was not significantly different from the pre-lesion response (*F*_6_=5.940, *P*=0.991). This recovery differed from that observed in the UVN-lesioned mice. Although the initial symptoms were quite similar to those of TTK-administered mice, the MORs never recovered to normal levels, even by 3 weeks after lesion induction (*F*_6_=35.398, *P<*0.001), and remained completely absent in 83% of the animals (Fig. 4F).

### Deafferentation of primary vestibular synapses evokes transient alterations in posturo-locomotor functions

**Fig. 5.**
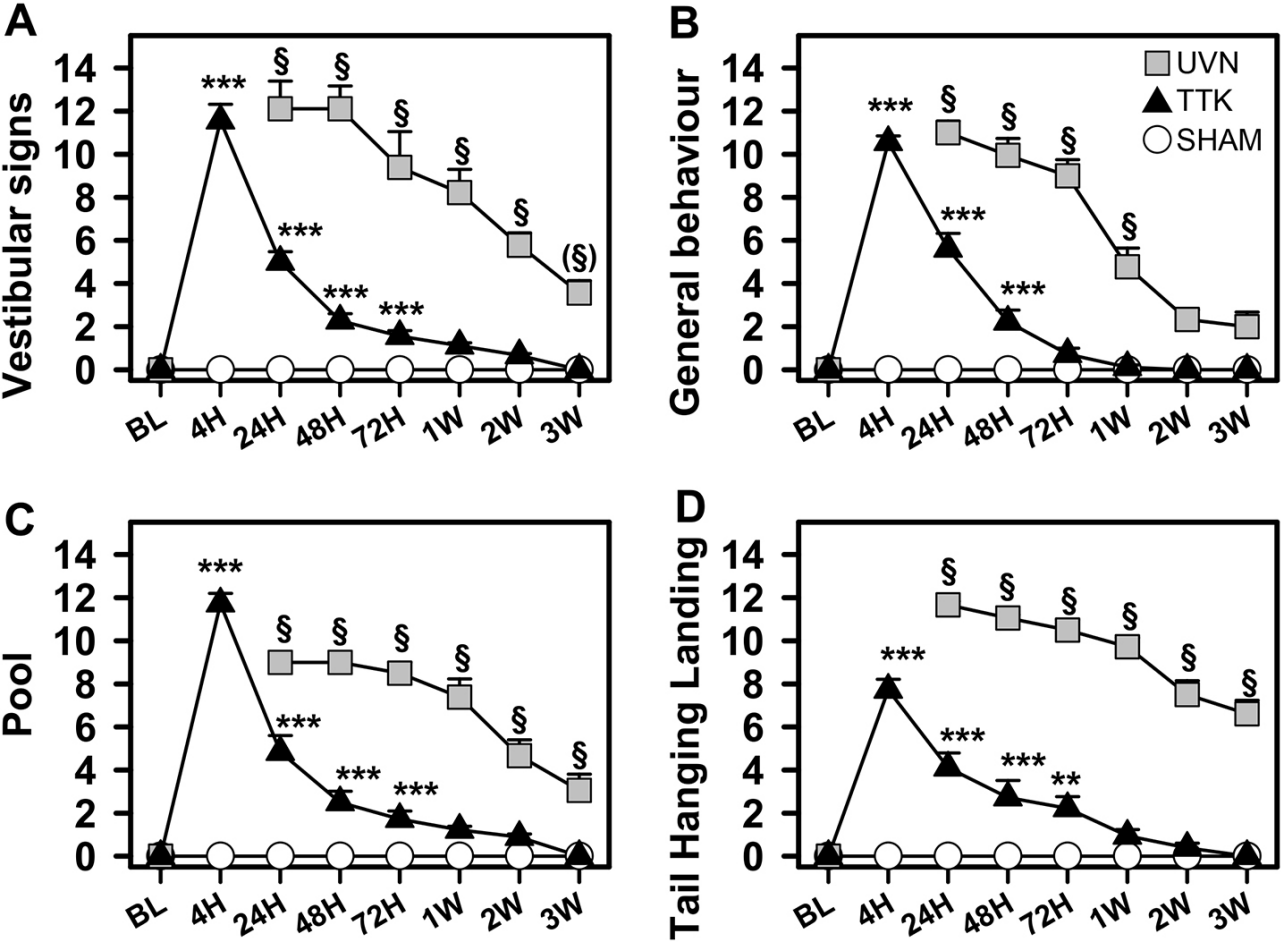
**Posturo-locomotor alterations and alterations of the general behaviour following TTK administration.** Evolution of vestibular alteration over time in TTK-treated, UVN-operated and sham mice during the 2 min evaluation. (A) Vestibular signs and (B) general behaviour in the open field. (C) Swim deficit score in the pool. (D) Postural alterations during the tail-hanging landing paradigm. Results are expressed as mean±s.e.m. The sham mice are represented by open circles, the TTK-treated mice by dark triangles and the UVN-operated mice by grey squares (*n*=9 in each group). Repeated measures ANOVA followed by Bonferroni post-hoc analyses were used to observe TTK administration effects (i.e. TTK vs sham mice): **P<0.01; ***P<0.001. We performed a repeated measures ANOVA followed by Bonferroni post-hoc analyses to observe treatment effects (i.e. TTK vs UVN): ^§^*P*<0.001, ^(§)^*P*=0.06.

A recently described battery of behavioural tests (Cassel et al., 2018) was used to assess the functional consequences of TTK administration on posturo-locomotor function and general behaviour (Fig. 5). Adult mice of the same strain that underwent UVN were also evaluated using the same paradigm to compare the consequences of the unilateral transient loss and the permanent loss of vestibular input. TTK administration evoked a dramatic and immediate increase in vestibular deficit symptoms (head tilt, muscle dystonia, circling, bobbing and barrel rolling; mean score±s.e.m.) that peaked at 4 h following lesion induction (11.5±0.76, *n*=9; Fig. 5A). The symptoms of vestibular deficits then progressively diminished until complete recovery 1 week after the lesion induction. A statistically significant effect of TTK administration (*F*_2,24_=89.67, *P*<0.001) and statistically significant differences between the TTK-administered and sham mice were observed up to 72 h after lesion induction (*P*<0.001). Alterations in the general behaviour (including vertical and horizontal exploration, body height and gait quality) followed similar kinetics (Fig. 5B). Statistically significant differences between the TTK-administered and sham mice were observed up to 48 h after lesion induction (*P*<0.001). The TTK-treated mice presented significant behavioural alterations (*F*_1,16_=292.11, *P*<0.001) that recovered over time (*F*_7,112_=123.17, *P*<0.001). The excitotoxic peripheral lesion also significantly impaired swimming ability. The swimming deficit score (see Materials and Methods section for details) transiently increased at 4 h (11.72±0.5, *n*=9; Fig. 5C) and then progressively decreased over the first week after lesion induction. Videos illustrating the swimming ability of the TTK-administered mice at 24 h and 2 weeks are provided (Movies 1 and 2). Statistically significant differences between the TTK-administered and sham mice were observed until 72 h after lesion induction (*P*<0.001). We found a global effect of TTK administration (*F*_1,16_=164.75, *P*<0.001) on this parameter. In the tail hanging landing (THL) paradigm, we observed a transient effect of the lesion (Fig. 5D). THL scores peaked at 4 h (7.72±0.5; *n*=9) and then progressively decreased over the first week. Statistically significant differences between the TTK-administered and sham mice were observed until 72 h after lesion induction (*P*<0.001). ANOVA analyses revealed a significant effect of TTK treatment (*F*_1,16_=73.58, *P*<0.001).

The UVN-lesioned mice also exhibited dramatic and immediate increases in posturo-locomotor function deficits and related behavioural changes (Fig. 5 grey squares). Compared with the TTK paradigm, the UVN protocol induced acute vestibular syndrome that was so severe that we could not perform behavioural testing at the 4 h time point, as the animals remained immobile, lying on their sides and sometimes barrel rolling. By 24 h after lesion induction, the mean vestibular deficit symptom score reached 12.11±1.27 (*n*=9). The recovery kinetics significantly differed from those observed in the TTK-administered mice. A reduction of 50% in the vestibular symptom deficit score was obtained 2 weeks after lesion induction, compared with 24 h after lesion induction in the TTK-administered mice (Fig. 5A). This score was still significantly different from that in the mice from the sham group 2 weeks after UVN induction (*P*<0.001). Similar results were obtained for swimming deficit scores (Fig. 5B), general behavioural deficits (Fig. 5C) and the syndrome reactivation in the THL paradigm (Fig. 5D). These results highlighted the difference in lesion severity and the difference between central compensation alone or central compensation plus peripheral repair.

### Correlation between the state of hair cell afferentation and the symptoms of the acute vestibular syndrome

**Fig. 6.**
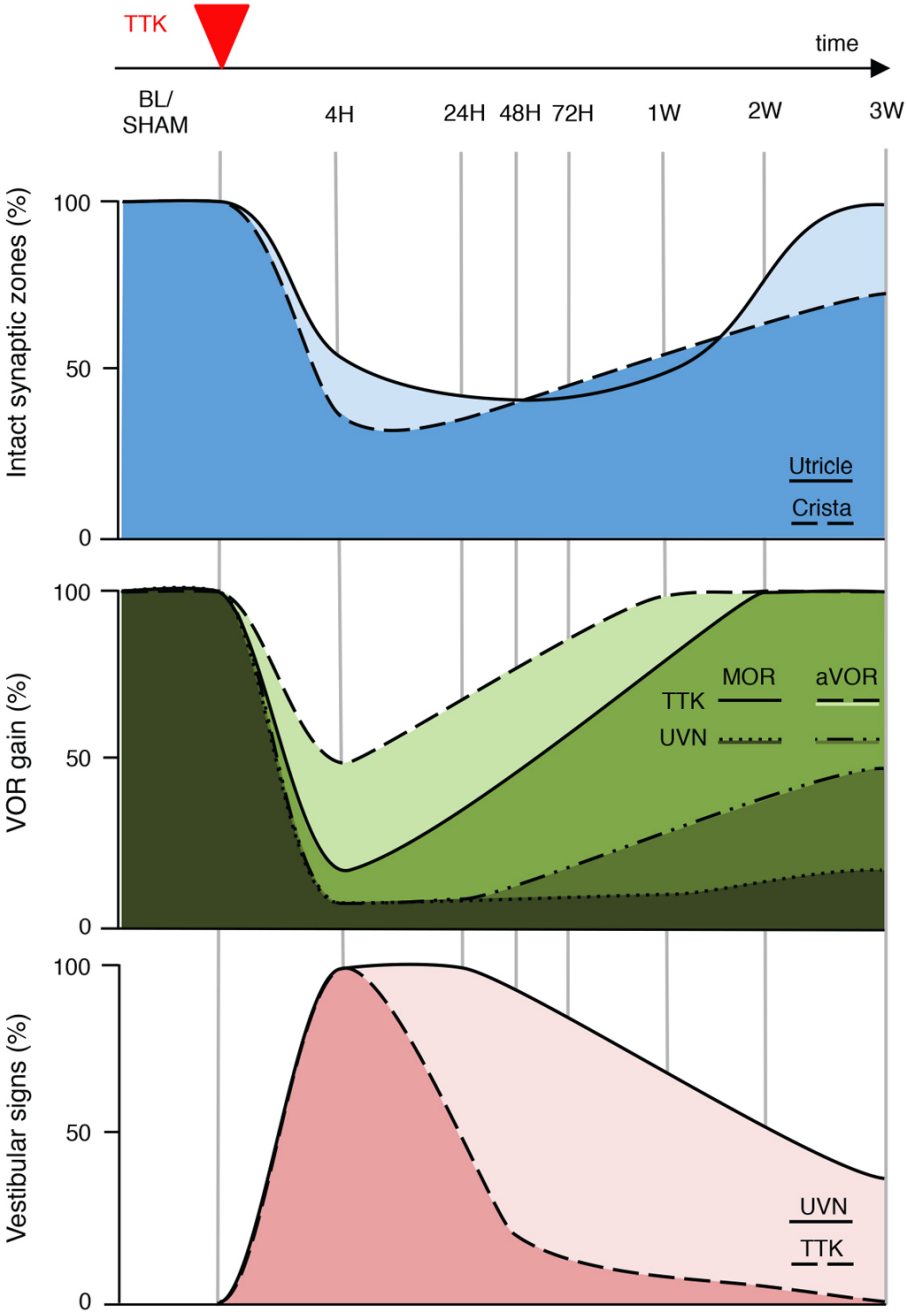
**Correlation grid between vestibular primary synapses afferentation state and signs of vestibular functional alterations.** Upper traces: schematic representation of the percentage of intact synapses in the utricle (light blue) and crista (deep blue), drawn on the basis of CtBP2 expression in the sensory epithelia at each of the investigated time points (Fig. 3). Middle traces: schematic representation of the percentage of aVOR [dashed line (or light green) for TTK and dash-dotted line (or lighter dark green) for UVN] and MOR [full line (or green) for TTK and dotted line (or darkest green) for UVN] gains, drawn on the basis of the aVOR measurements shown in Fig. 4. Lower traces: schematic representation of the percentage of posture-locomotor alterations in the UVN (light pink) and TTK (dark pink) mice, drawn on the basis of the vestibular signs displayed in Fig. 5A.

To better determine the temporal correlation between the tissue damage induced by TTK administration and its functional consequences, we established a correlation diagram for visualising the degree of synaptic damage within the two types of vestibular sensory epithelia, gaze stabilising alterations and posturo-locomotor deficits, in parallel (Fig. 6). For this purpose, we assumed that the percentage expression of CtBP2 in the utricle and the crista was representative of the percentage of intact synapses. Before excitotoxic lesion induction, 100% of the vestibular synapses within the selected observation fields in both the utricle and crista were intact. Under these conditions, both the aVOR and MOR gains were normal. No alterations in gait or balance were observed. Over the first hours following lesion induction, the maximum deafferentation of primary vestibular synapses (on the order of 67% in the utricle and 70% in the crista) was observed. The observed 60% and 82% reduction in the aVOR and MOR normalised gains (at 0.5 Hz) mirrored synaptic loss. Posturo-locomotor deficits peaked 4 h after lesion induction. Between 4 h and 24 h, before synaptic repair began, a massive restoration of aVOR gains and a marked reduction in vestibular deficit symptoms were achieved in the TTK-administered mouse model. During this period, functional recovery reached its maximal speed. By 24 h, both vestibulo-ocular and posturo-locomotor deficits recovered by half. Between 24 h and 1 week after lesion induction, the progressive regression of posturo-locomotor and vestibulo-ocular deficit symptoms continued, although the kinetics were different from those observed during the first 24 h. By 1 week after TTK administration, when the first changes in primary synapses were observed, the aVOR and MOR gains fully recovered. At this stage, the posturo-locomotor deficits and the resulting general behavioural alterations completely disappeared. In the UVN-lesioned mice, the aVOR also recovered, although this recovery remained incomplete (maximum recovery of 39% of the initial gain at 0.5 Hz). The MOR gain showed no recovery. Beyond 1 week after the lesion induction, the processes of synaptic repair were fully engaged in the TTK-administered mouse model. Both TEM and immunohistochemical analysis of CtBP2 and SHANK-1 expression at primary vestibular synapses indicated that synaptic repair was complete by 3 weeks after excitotoxic insult. Although the aVOR and posturo-locomotor deficits had already fully recovered in the TTK-administered mouse model, they remained only partially recovered at 3 weeks in the UVN-lesioned mice.

## DISCUSSION

Excitotoxicity is a pathological process that induces neuronal alteration and destruction through the massive release of glutamate and its analogues (Olney, 1969). When glutamate is released in quantities exceeding glutamate transport capacity, the overactivation of glutamate receptors causes the massive entry of cations into cells expressing these membrane receptors. In synaptic terminals, this ion influx triggers both a cascade of cellular mechanisms leading to structural damage (such as cytoskeleton damage and DNA degradation) and a massive inflow of water that can cause swelling and subsequent synaptic uncoupling (Pujol and Puel, 1999). Many observations have suggested that such phenomena might be involved in various auditory and vestibular pathologies; however, there is still no reliable histopathological evidence of inner ear excitotoxic damage associated with functional impairments in humans. By contrast, the involvement of excitotoxicity in the destruction of the inner ear primary synapses under conditions of ischaemia (Puel et al., 1994), overstimulation (Spoendlin, 1971) or ototoxicity (Smith, 2000) has been confirmed in various animal models. We have previously demonstrated that the administration of the glutamate receptor agonist kainate to the middle ear of rodents causes excitotoxic lesions and that these lesions are prevented in the presence of specific AMPA-kainate receptor blockers (Brugeaud et al., 2007). Kainate, which is not recycled by the glutamate transporters expressed at vestibular synapses (Bonsacquet et al., 2006), exacerbates the excitotoxic response and facilitates the study of functional consequences.

This study was designed to document the time course of an excitotoxic lesion development in the peripheral vestibular system at the histological and molecular levels and to understand how the state of synaptic connectivity correlates with the functional assessment of acute and subacute vertigo-associated deficits. TEM confirmed that the delivery of kainic acid to the inner ear in our model causes characteristic signs of excitotoxic lesions, such as the swelling and retraction of nerve endings, similar to those previously reported in rat models (Brugeaud et al., 2007; Dyhrfjeld-Johnsen et al., 2013; Gaboyard-Niay et al., 2016).

We demonstrated that the distribution of CtBP2- and SHANK-1-staining matches the previously reported distribution of CtBP2- and Glu2A2/3-staining (50% of the mean distances between CtBP2- and GluA2-positive staining were within 1 μm, 70% were within 2 μm and 100% were within 4-5 μm) in the crista of young (younger than 21 days old) rats (Sadeghi et al., 2014). This observation confirms the lack of juxtaposition between afferent ribbons and postsynaptic glutamate receptors. This distribution pattern largely relies on the particular organisation of the postsynaptic region of the calyx terminal (Wersall, 1956; Lysakowski and Goldberg, 1997; Desai et al., 2005a,b; Moser et al., 2006; Sadeghi et al., 2014), in which the PSD is especially elongated and does not fully face the presynaptic ribbons. We also confirmed that TTK administration causes the selective deafferentation of vestibular hair cells. This deafferentation is determined, on the one hand, by a significant increase in the distances between the pre- and postsynaptic proteins and, on the other hand, by a reduction in protein expression. Whether this loss results from a downregulation of protein expression requires further molecular investigations. CtBP2 and SHANK-1 expression was reduced by up to 65% in both utricle and crista hair cells and in afferent terminals between 4 h and 48 h after excitotoxic lesion induction. A similar phenomenon has already been reported for CtBP2 and GluA2/3 at auditory synapses following overexposure to sound (Liberman and Liberman, 2015). The high propensity of primary vestibular neurons to restore synaptic contacts with hair cells has been previously reported *in vivo* in rat models of excitotoxic-type vestibular lesions (Brugeaud et al., 2007; Dyhrfjeld-Johnsen et al., 2013; Gaboyard-Niay et al., 2016) and in rat and mouse models of chronic ototoxic exposure (Sedó-Cabezón et al., 2015; Greguske et al., 2018), as well as *in vitro* in organotypic co-cultures of Scarpa's ganglia and vestibular sensory epithelia (Travo et al., 2012).

In the TTK mouse model, TEM examination revealed that an initial phase of damage reduction and the restoration of the contacts between hair cells and afferent nerve fibres takes place within 1 week of the excitotoxic insult. Nevertheless, the observation of fragmented calyx endings indicated that the repair process was still underway. Similar discontinuities in calyx endings have been observed in premature calyces in late developmental stages (Desmadryl and Sans, 1990) and in other models of calyx-ending damage and repair (Seoane et al., 2001; Sedó-Cabezón et al., 2015). Therefore, these calyx endings were probably in a late stage of regenerative repair. In a second phase of repair that lasts another 2 weeks, the progressive upregulation of CtBP2 and SHANK-1 and subsequent synaptic pairing also takes place. Although only electrophysiological investigations using single fibre recordings can confirm the functional restoration of primary synapse activity, histological observations demonstrated that primary vestibular synapses undergo spontaneous repair following selective deafferentation. A further examination of the expression kinetics of the glutamate receptor subunits is required to confirm that the reported changes in the expression of the SHANK-1 also apply to the glutamate receptors.

The parallel analysis of histological and functional changes that occur over the different phases of vestibular syndrome in the TTK mouse model produced interesting information regarding their correlation. For example, the proportional reduction in aVOR gains and synaptic loss in the first hours following excitotoxic lesion induction suggests that the aVOR gain reductions observed immediately after lesion induction are directly indicative of the severity of vestibular sensory epithelium deafferentation. Over the first 24 h, the massive restoration of the aVOR gains and the marked reduction in vestibular signs are independent of synaptic repair, which begins several days later. This functional recovery can therefore be attributed to the reactive compensation processes that takes place in the brainstem vestibular nuclei (Lacour and Tighilet, 2010; Sadeghi et al., 2010). It can be assumed that the preservation of part of the nerve fibres connected to vestibular sensors is sufficient to feed the vestibular nuclei with information from the damaged vestibule and quickly diminish the imbalance of electrical activity considered to be the neurophysiological substrate of vertigo syndrome (Curthoys and Halmagyi, 1995; Dieringer, 1995).

One can then query the functional relevance of the peripheral synaptic repair if functional recovery is fully achieved before synaptic repair starts. The different symptoms that constitute the acute vestibular syndrome compensate each other with their own kinetics. Some of them, such as the head tilt, are slower than others (Dyhrfjeld-Johnsen et al., 2013; Cassel et al., 2018). It cannot be excluded that this specific parameter depends, at least partially, on peripheral synaptic repair. It is likely that the ability to spontaneously reform functional synapses is preserved at adult stage, according to the concept that ‘repair is better than compensation’. The feedback from peripheral sensory inputs, depending on the time it occurs, will possibly perturb the establishment of compensation mechanisms in the UVN-lesioned mice. We also anticipate that, during this phase, the progressive increase in peripheral inputs induces readjustments to the changes that occur in the earlier phases of repair.

The differences in the kinetics of gait and balance recovery between the TTK-administered and UVN-lesioned mice might result from central compensation processes that differ according to the type of peripheral lesion. This has been previously well demonstrated in different cat models of unilateral vestibular lesions (Lacour et al., 2009). The restoration of posturo-locomotor and oculomotor functions of cats subjected to UVN, was significantly delayed compared with that in groups subjected to the unilateral and transient pharmacological block of vestibular input or the selective destruction of the vestibular sensors. It has since been established that, upon UVN, profound rearrangements that affect local neuronal excitability, neurotransmitter and neurotrophin release, as well as local inflammatory responses (Lacour and Tighilet, 2010; Liberge et al., 2010; Dutheil et al., 2013, 2016), take place within the brainstem vestibular nuclei.

In patients with AUV, the segmental loss of isolated branches of the vestibular nerve peripheral to Scarpa's ganglion, with or without the degeneration of the associated sensory epithelium, has been reported (Schuknecht, 1993; Rauch, 2001). Although very scarce, such observations strongly support a causal link between the selective deafferentation of vestibular sensors and the occurrence of acute vestibular syndrome. Such patients display static and dynamic postural impairments, and alterations in canal- and otolith-dependent aVOR gains were also observed in the TTK mouse model (Hotson and Baloh, 1998). The spontaneous functional restoration of the injured vestibule has been demonstrated using different clinical assessments of the vestibular function, such as the caloric test, vestibular-evoked myogenic potential and, more recently, the video head impulse test. Over the weeks and months following the onset of pathology, significant functional recovery is observed in all semicircular canals (SCC), although to varying extents. This is well illustrated by a recent study of vestibular neuritis patients evaluated both during the acute phase and 2 months later (Büki et al., 2017); in this study, follow-up examinations reported 55% functional recovery in the horizontal canal and 38% in both the anterior and inferior canals. The precise mechanisms underlying the loss of vestibular endorgan function and its spontaneous functional recovery remain to be established. Given the paucity of histopathological observations confirming direct damage to vestibular nerve branches, however, an intralabyrinthine source is increasingly favoured (Uffer and Hegemann, 2016; Hegemann and Wenzel, 2017).

One question that remains is whether the reafferentation pattern of repaired synapses reproduces the preinjury pattern. One can, for example, imagine that orphan nerve fibres might simply reafferent the nearest neighbouring hair cells. This question is important because one can envisage that abnormal reafferentation (or at least reafferentation not completely corresponding to the initial pattern) might be a source of scrambled sensory inputs during movement. This type of situation could contribute to the chronic disabilities and the clinical deficits that often persist several years after AUV (Mandalà and Nuti, 2009; Strupp and Brandt, 2009). The demonstration that synaptic reafferentation might take several weeks to be established in mice suggests that, if this process is conserved in humans, a broad therapeutic window is available for pharmacological interventions. The identification of the molecular effectors and pathways involved in the repair of inner ear synapses should pave the way for the future development of therapeutic approaches aimed at protecting the peripheral synapses and stimulating their repair. Such studies would benefit all patients suffering the peripheral loss of synaptic connectivity.

## MATERIALS AND METHODS

### Animals

We used C57Bl6/J adult mice (2-3 months old) of both sexes (Charles River laboratories, L'Arbresle, France). All animals were kept in standard animal cages under conventional laboratory conditions (12 h/12 h light-dark cycle, temperature 22±2°C, humidity 55±5%) with *ad libitum* access to food and water. Behavioural experiments were conducted during the light phase. Experimental protocols and animal care were in compliance with the institutional guidelines (council directive 87/848, October 19 1987, Ministry of Agriculture and Forestry, Veterinary Department of Health and Animal Protection) and international laws (directive 2010/63/UE609, 13 February 2013, European Community) and policies (personal authorisation #I-67 Université Louis Pasteur-F1-04 for R.C.). The procedures for behavioural experiments and aVOR recordings were approved by the Neuroscience Ethics Committee N°71 from the French National Committee on animal experimentation and by the ethical committee for animal research of the University Paris Descartes.

### TTK administration

Unilateral TTK administration was performed on a total of 66 adult mice of both sexes. Under deep isoflurane anesthesia, a myringotomy was performed using a microneedle (30 gauge) and 20 µl of 25 mM kainate solution (Abcam ab120100 dissolved in NaCl 0.9%, pH 7.4) was infused into the middle ear using a Hamilton microliter syringe and a syringe pump. The anesthetised mice were then kept on their side (with lesion side facing up) for 30 min to optimise bathing of the round window with the kainate solution. For the sham condition, mice were administered NaCl 0.9% without kainate.

### UVN

UVN was performed on a total of 15 adult mice of both sexes following the surgical procedure previously reported for the rat (Péricat et al., 2017). Animals were anaesthetised with a mixture of ketamine 1000 (Virbac; 100 mg/kg, i.p)/methedomidrine (Domitor^®^ Orion Pharma; 0.2 mg/kg, i.p.). A tympanic bulla approach gave access to the vestibular nerve: cervical muscular planes were dissected leading to tympanic bulla, which was widely drilled to expose the stapedial artery and the promontory containing the cochlea. The cochlea was drilled exposing the cochlear nerve. The cochlear nerve meatus was enlarged with a needle leading to the vestibulocochlear nerve, which was sectioned, and the Scapa's ganglion aspirated. The wound was closed using a stapler. Before awakening the animal by intraperitoneal injection of Antisedan^®^ (Orion-Pharma; 1 mg/kg), a solution of Ringer Lactate (Virbac; 10 ml/kg) was administered subcutaneously to reduce the dehydration resulting from the inability to drink normally owing to the lesion. Buprecare^®^ (Axience; 0.005 mg/kg) was given as postsurgery analgesic. In another six sham mice, surgery was limited to the opening of the tympanic bulla.

### Light microscopy and TEM

Light and TEM observations were performed as described (Greguske et al., 2018) in a total of nine adult mice of both sexes (three mice in each group): sham (NaCl-injected mice corresponding to the control group), 4 h following TTK administration and 1 week following TTK administration. The temporal bones were immersed in cold 2.5% glutaraldehyde in 0.1 M cacodylate buffer (pH 7.2) and the vestibular epithelia were dissected in a fume hood within 5 min of sacrifice. The specimens were fixed for 1 h in the same fixative, rinsed with buffer, post-fixed with 1% osmium tetroxide in cacodylate buffer for 1 h, rinsed and then stored at 4°C in 70% alcohol. Afterwards, the specimens were dehydrated with increasing concentrations of ethanol (up to 100%) and then embedded in Spurr resin. Semi-thin sections (1 µm) were stained with 1% Toluidine Blue and examined under a light microscope. Ultra-thin sections were stained with uranyl acetate and lead citrate and were observed with a JEOL 1010 TEM microscope at 75-80 kV.

### Immunostaining of synaptic proteins

Immunolabelling experiments were performed using 45 adult mice of both sexes (6 mice at 4 h, 48 h, 72 h, 2 weeks and 3 weeks; 5 mice sham, 24 h and 1 week). Sham mice were NaCl-injected mice and correspond to the control group. The mice were deeply anaesthetised using intraperitoneal injection of pentobarbital (60 mg/kg). Their temporal bones were rapidly removed, and the vestibular epithelia were microdissected and fixed for 1 h at room temperature in 4% paraformaldehyde dissolved in PBS. The samples were stored at −20°C in a cryoprotectant solution (34.5% glycerol, 30% ethylene glycol, 20% PBS, 15.5% distilled water) until staining. The immunohistochemical analyses were performed on whole vestibular sensory epithelia (crista and utricle). Tissues were first incubated for a 15 min period in citrate sodium buffer (pH 6, 10 mM) at 95°C and then left for 90 min in PBS with 4% Triton X-100 and 5% donkey serum under slow agitation. The primary antibodies were incubated in 0.1% Triton X-100 and 1% donkey serum in PBS for 48 h at 4°C: mouse IgG1 anti-CtBP2, BD transduction #612044 (1/500); rabbit anti-SHANK1, Novusbio #NB300-167 (1/500); and goat anti-NKA α3, Santa Cruz #sc-16052 (1/750). We immunolabelled SHANK-1 rather than GluA2/3, because of the lack of reliability in our hands of anti-GluA2/3 antibodies in the vestibular tissues. After three washes, the secondary antibodies were incubated in 0.1% Triton X-100 in PBS overnight at 4°C: Alexa Fluor 488 donkey anti-rabbit IgG H&L (1/500) #ab150073; Alexa Fluor 568 donkey anti-mouse IgG H&L (1/500) #ab175472; Alexa Fluor 647 donkey anti-goat IgG H&L (1/500) #ab151031. A chromatin staining with DAPI was performed between the two final washes. The epithelia were oriented and mounted in Mowiol medium.

### Acquisition of fluorescence images

Fluorescence images were acquired using a Zeiss (LSM 710 NLO) confocal microscope with a 63× Zeiss Plan Apochromat oil-immersion lens (NA 1.40) controlled with the Zen software (ZEN 2012 Black edition, Carl Zeiss Microscopy GmbH, Germany). We acquired *z*-stacks (20-50 optical sections) through the majority of preparations that we collected in 0.40 µm steps. The step size (optical section thickness) was determined by stepping at half the distance of the theoretical *z*-axis resolution. Images were acquired to a resolution of 1024×1024 pixels at subsaturating laser intensities for each channel.

### Quantitative assessment of pre- and postsynaptic proteins

Quantitative analysis of CtBP2 (RIBEYE) and SHANK-1 spots was performed using IMARIS software (version 8.2.1) based on raw image stacks. We did not apply any deconvolution, filtering, gamma correction or resampling. We automatically detected the total number and relative location of CtBP2 and SHANK-1 puncta using the Spots function in the IMARIS 3D image visualisation. To evaluate the number of colocalisations/juxtapositions between CtBP2 and SHANK-1 proteins, an isosurface of each signal was created in independent colour channels (488 and 568 nm). Puncta volumes were then computed using IMARIS software functions that provide 3D rendering and visualisation of isosurfaces enveloping all pixel clusters. Puncta volumes were computed along the *x*, *y* and *z* coordinates of their centres. Automatically detected puncta were verified by eye in a rotary 3D reconstruction of the sample. Puncta that were not contained within or next to the hair cells were manually removed. The analysis software MatLab (The MathWorks, Natick, MA) was used to set the colocalisation distance threshold at 1 µm in vestibular end organ samples. We used a custom R software Script (R Foundation for Statistical Computing, Vienna, Austria) to calculate the distances between CtBP2 and SHANK-1 labels from the *x*, *y* and *z* coordinates.

Considering there were fewer CtBP2 than SHANK-1 spots, we assessed the distances from CtBP2 to SHANK-1 and vice versa (see Figs S3 and S4). Quantitative analyses were performed on observation fields of 4.5e^−03^ mm^2^ (observation field in Fig. 3), which include about 75-100 hair cells in both utricles and crista for each of the five to six animals used per time point. In Fig. 3 and Table 1 the ribbon counts were normalised relative to the observation field. We could not estimate the volume of the observation fields, because the *z*-axis lengths differed in function of observed tissues.

Note that the calculated distances between CtBP2 and SHANK-1 immunolabelled spots do not necessarily reflect absolute distances, owing to deviations that probably result from the inherent limits of resolution imposed by immunofluorescence and confocal microscopy.

### Video-oculography and vestibulo-ocular procedures

Video-oculography was performed to quantify vestibulo-ocular reflexes. A total of 27 C57Bl6/J mice of both sexes were subjected to either TTK, UVN or equivalent sham conditions. All surgical and pre-test procedures were similar to those previously described (Péricat et al., 2017). Briefly, mice were head-fixed in a custom-built Plexiglas tube secured on the superstructure of a vestibular stimulator. The aVOR were tested in complete dark (light intensity <0.02 lux). Eye movements were recorded using an infrared video system (ETL-200, ISCAN, Burlington MA). Eye and head position signals were sampled at 1 kHz, digitally recorded (CED power 1401 MkII) with the Spike 2 software and later exported into the Matlab programming environment for off-line analysis (Matlab, The MathWorks). To maintain miosis throughout the experiment, 2% pilocarpine was applied 10 min before the start. Gaze stability was tested in the dark by recording spontaneous eye movements in the absence of any vestibular stimulation. Then, aVOR was tested during horizontal sinusoidal rotation of the turntable (0.2-1 Hz; peak velocity 30°/s). To specifically test otolithic function (MOR), the turntable was tilted by 17° and off-vertical axis rotation (OVAR) was performed (50°/s constant velocity; in clockwise and counter-clockwise direction). Analysis was performed off-line. Horizontal and vertical eye and head movement data were digitally low-pass-filtered (cut-off frequency 40 Hz), and position data were differentiated to obtain velocity traces. Segments of data with saccades were excluded from aVOR slow-phase analysis. Details of the analysis have been previously reported (Beraneck and Idoux, 2012; Carcaud et al., 2017).

### Behavioural exploration

Behavioural evaluation of the posturo-locomotor function was performed in a group of 27 adult mice subjected to either TTK, UVN or sham conditions. We used three paradigms: the open field (OF), the tail-hanging landing (THL) and the swim test. For the OF and THL, a 30×30 cm square of Plexiglass fixed on a bottomless table was used. A camera was placed under the open field to monitor mouse behaviour from below. In the THL test, the mice were gently held by the tail and quickly moved vertically back and forth along a 50 cm long path. We used a Plexiglass box (length 34 cm, width 21 cm and height 20 cm) filled with water (maintained at 32°C to avoid hypothermia) as a pool for the swim test. Before surgery, a 5 day handling period was implemented to habituate the animals to manipulation (2 min per day for each mouse). We assessed mouse behaviour before surgery (base line, BL) and at the following time points thereafter: 4 h, 24 h, 48 h, 72 h, 1 week, 2 weeks and 3 weeks. The items were quantified in a scale from 0 (mice exhibited no deficits at all), to 3 (the highest degree of vestibular deficit). Briefly, in the OF, we recorded spontaneous animal behaviours over 2 min and quantified two sets of parameters: we first looked at items linked to specific vestibular deficit symptoms and scored behaviour relative to mouse global state (referred to as general behaviour). Signs of vestibular deficits included specific symptoms such as circling (stereotyped movement in circles around the hips of the animal), head tilt (inclination of the head in the roll plane), muscle dystonia (hypertonia on the lesioned side), barrel rolling (mice turning on themselves around their longitudinal axis) and head bobbing (abnormal intermittent flexion-extension of the neck in the pitch plane). General behaviour encompassed the quality of horizontal and vertical exploration, quality of locomotion and centre of gravity height. During the THL procedure we evaluated the quality of forelimb extension normally produced to reach the ground (i.e. quality of the landing) and the body axial rotation (twirl). We also measured the intensity of the syndrome reactivation after landing as the accentuation or the reappearance of vestibular deficit symptoms (circling, head tilt, muscle dystonia, bobbing) from 0 (no sign) to 3 (maximum expression/accentuation of vestibular deficit symptoms). The swimming abilities of the animals were then quantified for 30 s (cut-off time) by observing the quality of swimming (specific grid detailed in Cassel et al., 2018) and whether the mice were barrel rolling or not in the water. Once mice were removed from the water, we observed the intensity of syndrome reactivation and their grooming skills.

### Statistics

Immunostaining counting and processing were analysed using one-way analysis of variance (ANOVA). When appropriate, the Dunnett multiple comparison test followed ANOVA. All data are reported as mean+s.e.m. Values of *P<*0.05 were considered significant.

For the aVOR and OVAR analyses, when considering the factor ‘time’, comparing values to the before-lesion values, significance was measured using a repeated measures ANOVA: two-way for aVOR values (time and frequency) and one-way for OVAR (time). When comparing values between UVN and TTK populations, significance was measured using a three-way (time, frequency and lesion type) or two-way (time and lesion type) ANOVA. Post-hoc comparisons were performed using the Tukey HSD test. All data are reported as mean±s.e.m. Values of *P*<0.05 were considered significant. We report the lack of statistically significant effects by the letters n.s.

For behavioural exploration, performances recorded across the different paradigms were analysed using one-between, one-within factor ANOVA, which considered the factors ‘time’ (time points 1-8; i.e. BL, 4 h, 24 h, 48 h, 72 h, 1 week, 2 weeks and 3 weeks) and ‘treatment’ (vehicle vs TTK) or (vehicle vs UNV). The ANOVA was followed by a Bonferroni multiple-comparisons test when appropriate. For all ANOVAs reported, violations of the sphericity assumption (homogeneity of covariance) were corrected using the Greenhouse-Geisser procedure; the corrected *P*-value along with the epsilon correction factor (ε) are reported. As ε was lower than 0.75 in all analyses, we used G-G corrected *P*-value for all data presented. Results are expressed as mean±s.e.m. Values of *P*<0.05 were considered significant. We report the lack of statistically significant effects by the letters n.s.

## Supplementary Material

Supplementary information
