# Supplementary material for: Morphological and functional correlates of vestibular synaptic deafferentation and repair in a mouse model of acute-onset vertigo
Source: Dis Model Mech. 2019 Jul 15;12(7):dmm039115. doi: 10.1242/dmm.039115 (PMC6679379; doi:10.1242/dmm.039115)
Supplement: Supplementary information [file dmm-12-039115-s1.pdf]

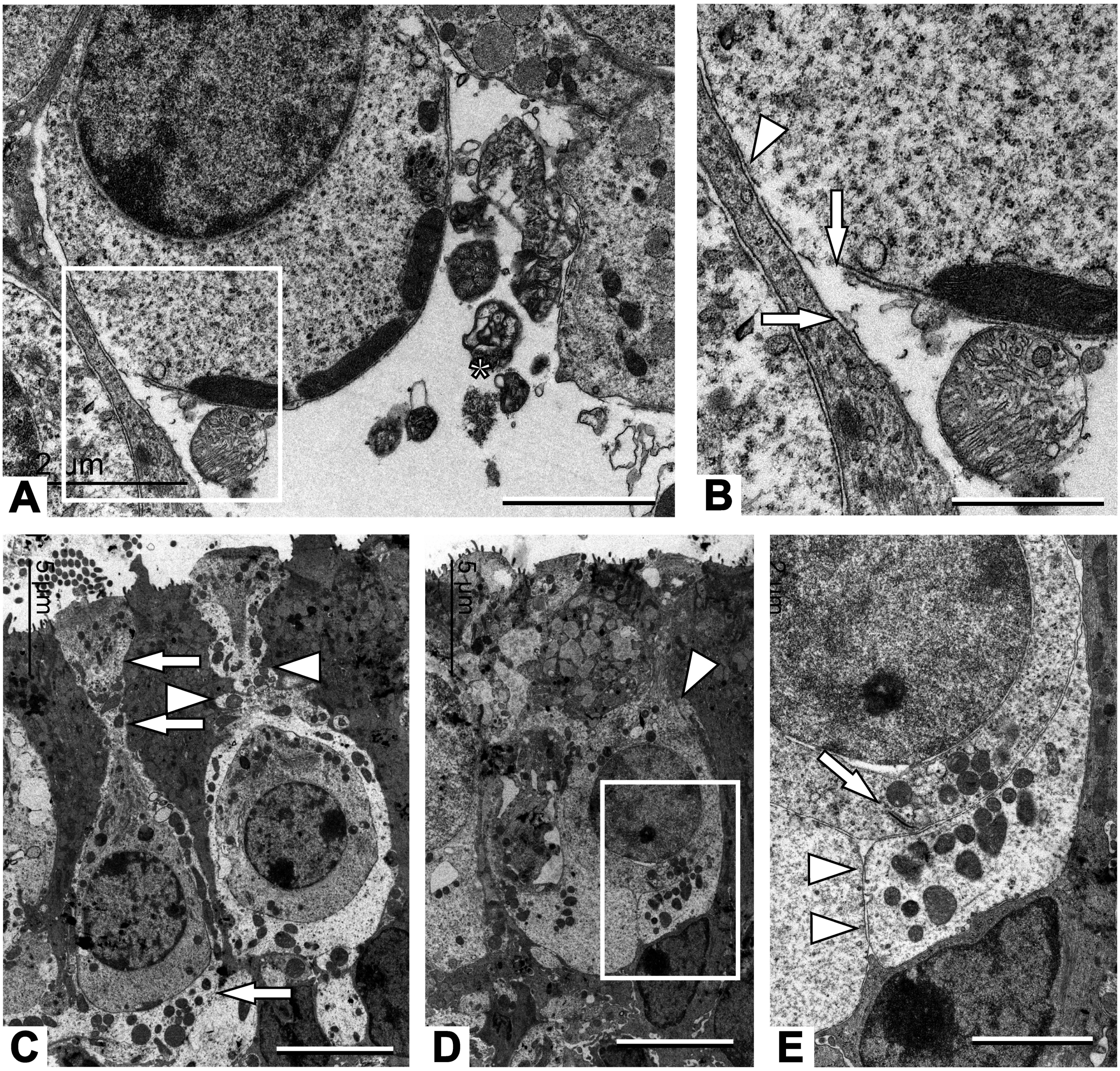

Figure S1: TEM observation of vestibular primary synapses in the mouse utricle following TTK. (**A and B**) Four hours following the TTK administration, a swollen calyx ending contacts a type I hair cell (A). The boxed area in A is shown at higher magnification in B. Disruptions in the hair cell and calyx membranes are clearly identifiable (arrows in B). Mitochondria display modified ultrastructure in the calyx afferent (* in A). (**C**) Two type I hair cells at one week after TTK. A calyx afferent displaying normal ultrastructure (arrows), encases the cell on the left. In contrast, the calyx ending surrounding the right cell shows a fragmented appearance in the neck region of the cell (arrowheads). (**D**) A type I hair cell with an incomplete and fragmented calyx. Note the upper end of the calyx, falling short of the position it should attain closer to the apical end of the cell. Note also the fragmented nature of the afferent in the

basal part of the cell (box). (**E**) This higher magnification of the area boxed shows the fragmented nature of the calyx ending (arrowheads). Nevertheless, a ribbon (arrow) is clearly observed in the pre-synaptic side. Sample: n = 3 in each group. Scale bars= 2 µm in A and E; 1 µm in B; and 5 µm in C and D.

Disease Models & Mechanisms • Supplementary information

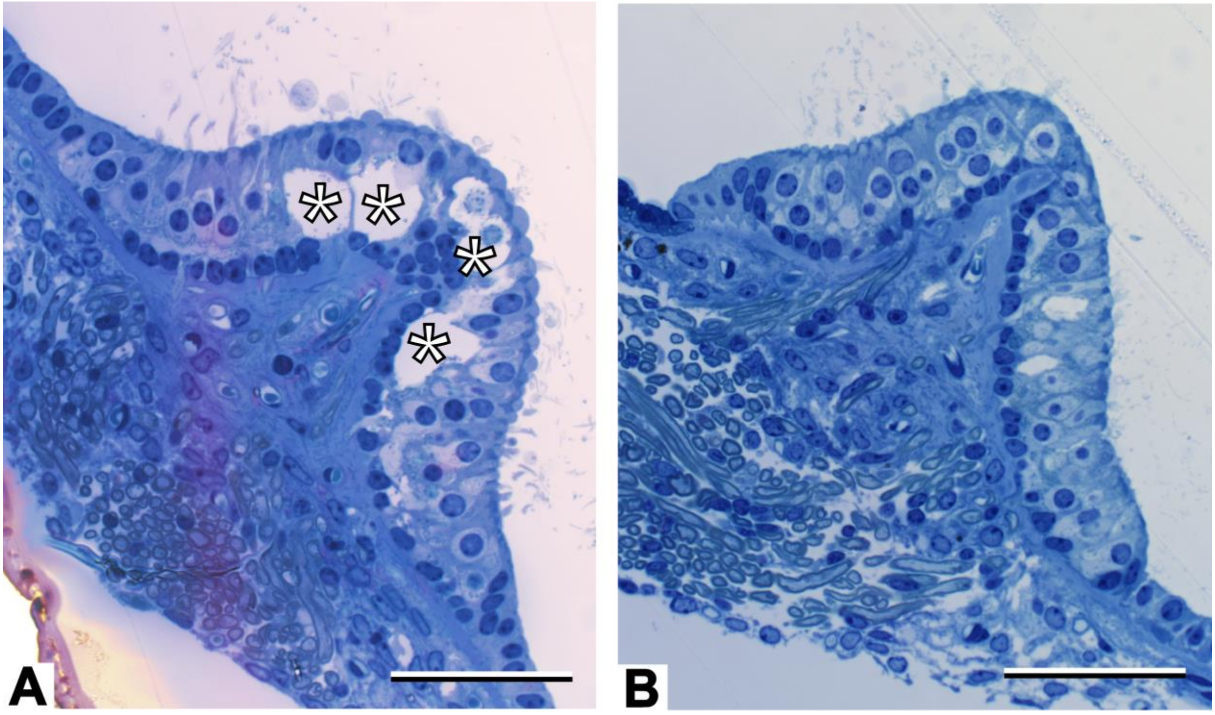

Figure S2: Light microscopy observation of vestibular crista following TTK. (**A**) At 4 h after TTK, large vacuoles are observed throughout the crista sensory cells layer. The swellings were often more visible in the central part of the crista (*). (**B**) A representative crista one week after TTK administration, showing control-like morphology. Sample: n = 3 in each group. Scale bars= 50 µm.

Disease Models & Mechanisms • Supplementary information

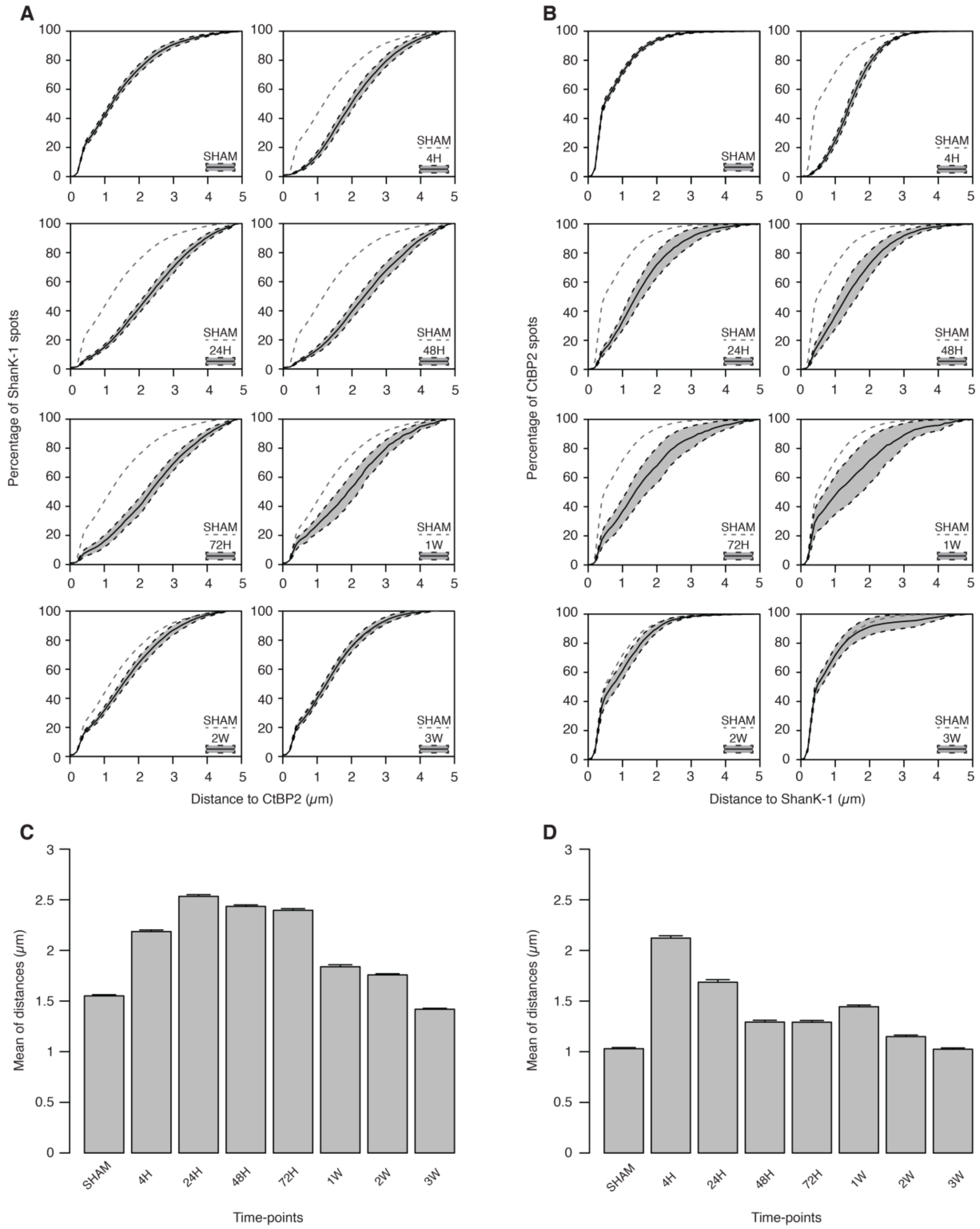

Figure S3: Time dependent variation of cumulative and mean distances between CtBP2 to SHANK-1 and vice versa following TTK administration in the crista. Variation of cumulative distances (**A and B**) and mean distances (**C and D**) at each selected time points. (n = 6) for 4h, 72h and 3W; (n = 5) for Sham, 24h, 48h, 1W and 2W. Results are expressed as mean ± SEM.

Disease Models & Mechanisms • Supplementary information

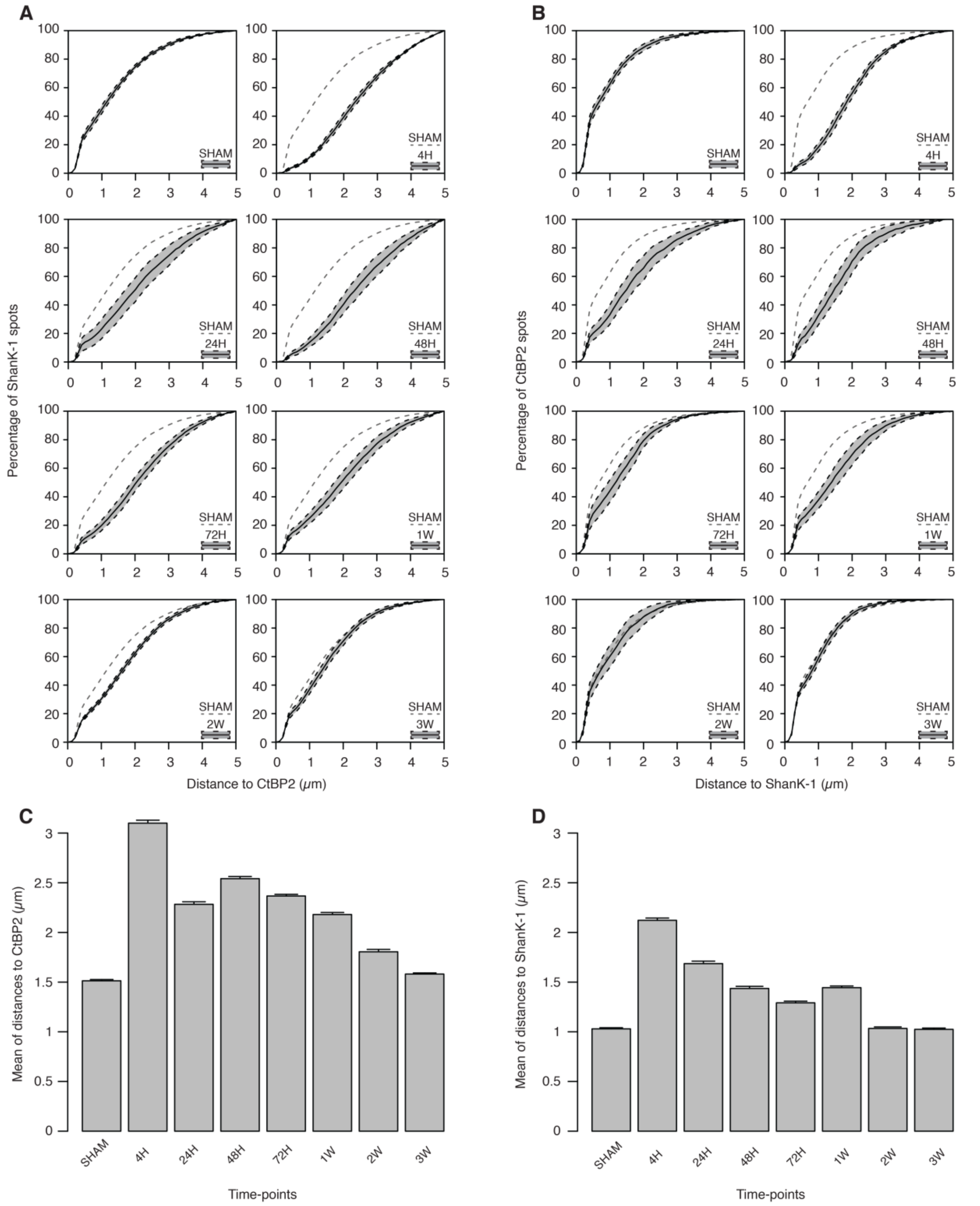

Figure S4: Time dependent variation of cumulative and mean distances between CtBP2 to SHANK-1 and vice versa following TTK administration in the utricle. Variation of cumulative distances (**A and B**) and mean distances (**C and D**) at each selected time points. (n = 6) for 48h, 72h and 2W; (n = 5) for Sham, 24h and 3W; (n = 4) for 4h and 1W. Results are expressed as mean ± SEM.

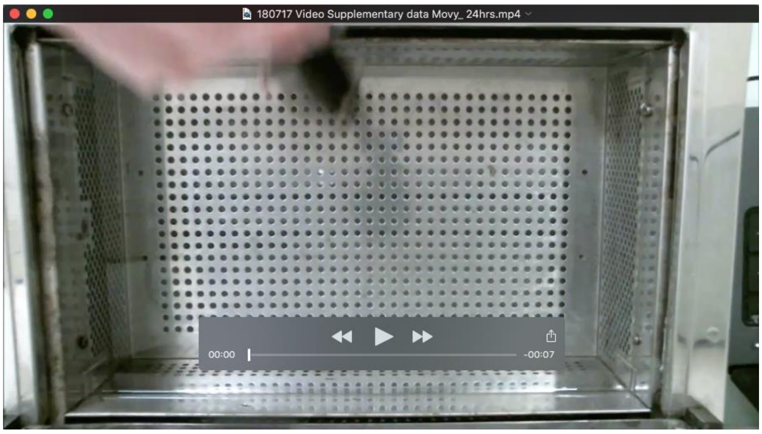

Movie 1: This video illustrates the loss of the swimming ability in a mouse at 24h after the TTK administration.

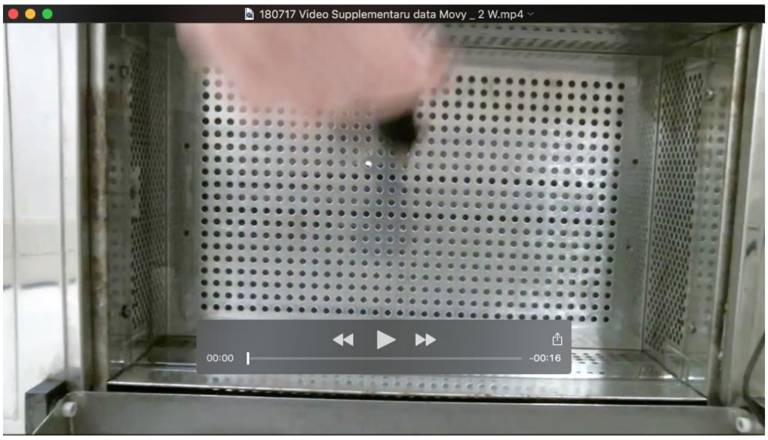

Movie 2. This video illustrates the recovery of the swimming ability in a mouse at 2 weeks after the TTK administration.

Disease Models & Mechanisms • Supplementary information
